# Identifying the predictors of turnover intention based on psychosocial factors of nurses during the COVID‐19 outbreak

**DOI:** 10.1002/nop2.896

**Published:** 2021-05-07

**Authors:** Alireza Mirzaei, Hamed Rezakhani Moghaddam, Aghil Habibi Soola

**Affiliations:** ^1^ Department of Emergency Nursing School of Nursing and Midwifery Ardabil University of Medical Sciences Ardabil Iran; ^2^ Department of Public Health Khalkhal University of Medical Sciences Khalkhal Iran; ^3^ Department of Nursing School of Nursing and Midwifery Ardabil University of Medical Sciences Ardabil Iran

**Keywords:** COVID‐19, general health, job content, post‐traumatic stress disorder, turnover intention

## Abstract

**Aims:**

Because of the direct contact nurses have with patients, they are exposed to more stressful events during the outbreak of infectious diseases, which increases their turnover intention, highly impacting not only nurses, but also patients and organizations. The present study aimed to identify the predictors of turnover intention based on psychosocial factors in nurses of Ardabil pre‐hospital emergency and educational and medical centres during the COVID‐19 outbreak.

**Design:**

The present descriptive‐analytical study was conducted in June, 2020.

**Methods:**

A total of 479 nurses working in Ardabil pre‐hospital emergency and educational and medical centres to fight COVID‐19 were recruited for this study using the census method. Data were collected using the Demographic Information Questionnaire, Turnover Intention Questionnaire, Weiss & Marmar Impact of Event Scale‐Revised (IES‐R), General Health Questionnaire (12 C‐GHQ) and Job Content Questionnaire (JCQ). Data were analysed with SPSSv.22 software using correlation, *t* test, analysis of variance, multiple regression and descriptive tests.

**Results:**

The mean turnover intention score of nurses was 41.73 with a standard deviation of 12.11. The results of correlation coefficient revealed a positive relationship between PTSD, general health, job demand and job strain with turnover intention (*p* ≤ .01) and a positive and significant relationship between social support and turnover intention (*p* ≤ .01). Multiple regression analysis showed that the variables of gender, marital status, work position, decision latitude, social support, job strain, general health and post‐traumatic stress disorder (PTSD) were predictors of turnover intention.

**Conclusion:**

Job stressors during the COVID‐19 outbreak have led to an increase in nurses’ turnover intention. Identifying and managing the factors related to job stressors will make it possible to prevent nurses’ turnover intention in such critical situations.

## 1 INTRODUCTION

After COVID‐19 was first observed in China in December 2019, this disease spread rapidly around the world (Wang et al., 2020), and the number of infected people is increasing. The outbreak of this virus has resulted in an unprecedented crisis in the health systems of many countries and a serious challenge to the international community. Policymakers in various countries have adopted different strategies to prevent and treat this disease, depending on the local prevalence of the virus, healthcare system resources, economic and political factors, and people's perception of the situation (Rossman et al., 2020). Iran reported the first confirmed cases of COVID‐19 infection in February, 2020 in Qom (Tuite et al., 2020). Other cities soon reported cases of COVID‐19, resulting in the closure of schools and universities in many provinces and the cancellation of numerous cultural, sports, and religious gatherings and conferences. The first case of COVID‐19 in Ardabil province was reported on March 28, 2019. Health recommendations were initiated to prevent further infection (Science, 2020), and in early June, a slightly downward trend of COVID‐19 was reported in Ardabil province (Agency, 2020). Health emergencies like the outbreak of COVID‐19 increase the need for communities to provide general health services. Nurses provide the core elements for the proper functioning of the healthcare delivery system in such situations (Charney et al., 2015), since they provide safe and sustainable patient care and play a key role in controlling the disease outbreak (Alipour et al., 2018). The increased number of patients and their need for intensive care in hospitals also increases the need for nurses' health services.

Previous research conducted on outbreaks of infectious diseases, such as severe acute respiratory syndrome (SARS), avian influenza (AV) and MERS Syndrome (MERS‐CoV), has shown that such outbreaks influence health care providers' interest in their jobs (Wong et al., 2010) and increases their turnover intention (Jung et al., 2020). Turnover intention is a person's willingness to leave the job for which he is currently employed. The desire to leave a job is synonymous with anticipated turnover, which means that a person is likely to leave a job position. The intention of nurses to leave the job leads to an increase in the cost of employment and training for relevant hospitals and is a waste of nursing training resources (Hariri et al., 2012). Investigating the factors influencing a nurse's turnover intention can help hospital managers take preventive measures and address potential problems in clinical nursing work that may cause a nurse to relocate (Chen et al., 2018) to attend to current concerns about nursing shortages. The turnover intention of nurses is an important issue. There is evidence that the low retention of nurses in healthcare practices is related to heavy workload, burnout and job dissatisfaction (Cai & Zhou, 2009). It is an issue that has affected many healthcare institutions for years. The mean turnover intention score varies from one country to another. In the Asian countries of Indonesia (Labrague et al., 2020) and South Korea (Lee, 2019), it has been reported at 15% and 25%, respectively, among Western countries such as the United States (Solutions & Inc, 2019), it is reported to be 18%, and in Iran (Sokhanvar et al., 2018), the mean turnover intention score of nurses is 32.7%. The loss of experienced nurses has a negative impact on the provision and continuity of patient care services and may result in more adverse events, lost nursing care and patient mortality (Griffiths et al., 2019). These conditions are more evident during outbreaks of viral diseases; turnover intention was reported more during outbreaks of SARS and COVID‐19 (De los Santos & Labrague, 2020; Jung et al., 2020). The results of recent studies have suggested a significant relationship between turnover intention and PTSD in nurses involved in patient care during the outbreak of MERS (Jung et al., 2020). Since nursing staff are exposed daily to stressful events, including violence, patient deaths, emergencies and critically ill patients, they are more likely to suffer post‐traumatic stress symptoms (Lavoie et al., 2011). PTSD during a disease outbreak is estimated to affect up to 8% of the total population (Ebrahimpour et al., 2017), but during the SARS outbreak, 57% of nurses showed symptoms of PTSD (Jung et al., 2020), and during the COVID‐19 outbreak, the overall mean of PTSD was 1.78 (range of scores was 1 to 3; Nowicki et al., 2020). Symptoms usually last for more than a month and affect important aspects of one's life, such as the quality of one's professional life and patient care (Saraji & Dargahi, 2006). Like other known disorders in the area of mental pathology, PTSD endangers the physical and mental health of the affected person (Selaman et al., 2014). Desirable general health is considered as one of the factors inhibiting the negative effects of job stress. Nurses face various stressful situations in their profession, which might cause problems such as dissatisfaction, reduced efficiency and turnover in nurses. These problems cause irreparable damage on the provision of clinical care to patients (Shahraki Vahed, 2010). Moreover, psychological distress leads to more fatigue, less job satisfaction, and an increased tendency to leave the job (DeTienne et al., 2012).

Job stress is one of the factors that threaten nurses' general health, reduce their performance, and provide the conditions for turnover by increasing their job burnout (PEIMAN et al., 2013). It also affects job satisfaction and organizational commitment of staff members and is a predictor of turnover intention. It should be noted that nursing is a stressful and risky job. Nurses often face critical events and acute risk factors (Jafari et al., 2017). There are two models that show how social support affects stress outcomes. The direct‐ (main‐) effect model and the buffering (moderating) model. First, mere social support, whether or not the individual is under the influence of stress, causes the individual to avoid negative experiences, and therefore has beneficial effects on health. The direct‐impact model suggests that social support, regardless of stress, has a positive effect on various well‐being outcomes (Cohen & Wills, 1985). The second theory states that social support acts as a moderator against the effects of high stress on well‐being and related outcomes. Likewise, social support reduces the negative consequences of stress such as health disorders, anxiety, depression, burnout and turnover intention. This model states that social support protects employees against the pathological consequences of stressful experiences (Brouwers et al., 2001; Cohen & Wills, 1985; Väänänen et al., 2003). Different sources of social support can moderate the effects of stress on health in different ways (Väänänen et al., 2003). In fact, having social support, especially the support of co‐workers, is one way to deal with job stressors (Brouwers et al., 2001).

Many factors, such as social aspects, job satisfaction, wage and income level, appropriate relationships with co‐workers, and supervisor support, are among the factors that affect turnover intention. Having appropriate relationships, especially adequate support at the workplace, is a successful management strategy for reducing turnover intention after the spread of epidemics (Jung et al., 2020). Studies have indicated that nurses who have turnover intention have lower decision latitude and social support and have more psychological and physical needs and job insecurity (Barzideh et al., 2013). Turnover intention among nurses during the spread of infectious diseases can have serious impacts on the maintenance and provision of adequate health services. The current study aimed to evaluate the relationship between turnover intention and job stressors in nurses of pre‐hospital emergency and educational and medical centres during the outbreak of COVID‐19.

## 2 METHODS

The present descriptive‐analytical study was conducted in June 2020 after the first peak of COVID‐19 disease in the white^1^ situation of Ardabil, an ancient city in northwestern Iran and the capital of Ardabil Province. The statistical population of the study included nurses employed in Ardabil pre‐hospital emergency and educational and medical centres who were first line providers of care for COVID‐19 patients. A convenience sampling method was used in this study. Inclusion criteria of the study were more than six months of clinical work experience and a willingness to participate in the study. Exclusion criteria comprised employment in hospital departments not involved with COVID‐19 and incomplete questionnaires. After obtaining approval for the research plan and a license to conduct research from the Research Ethics Committee of Ardabil University of Medical Sciences (IR.ARUMS.REC.1399.035), sampling was performed. The researchers introduced themselves through e‐mail to nurses who met the inclusion criteria and explained the study's purpose and procedure, nurses were assured of the confidentiality of the data, and their consent to participate was obtained. The questionnaires were sent online to 765 nurses who met the inclusion criteria; 479 nurses completed their questionnaires without providing their names.

### 2.1 Research tools

Data were collected through demographic and occupational information forms, the impact of event scale‐revised, the 12‐item general health questionnaire, Kim et al.’s turnover intention questionnaire, and the job content questionnaire (JCQ). The demographic and occupational information form included age, gender, marital status, educational status, work position, work experience, department of service and monthly income. The Impact of Event Scale‐revised was developed by Weiss and Marmar (Weiss & Marmar, 1997) according to the DSM‐IV criteria for diagnosis of PTSD (Weiss, 2007). The Persian version of this tool has been validated in Iran (Panaghi et al., 2006). The IES‐R questionnaire includes 22 items, 8 of which are related to avoidance symptoms, 8 items to intrusion, and 6 items to hyperarousal symptoms. Participants rate the frequency of experiencing each symptom over the preceding seven days on a scale including 0 (never), 1 (rarely), 2 (sometimes), 3 (often) and 4 (usually). Scores range from 0–88 with higher scores indicating higher stress levels. The Cronbach's alpha value in the study conducted by Weiss and Marmar was 0.79 (Weiss, 2007), and in the present study was 0.86. The General Health Questionnaire (GHQ) was designed as a self‐administered screening test for detecting, and measuring, minor psychiatric disorders or psychological distress (Goldberg, 1978, 1988). This scale consists of 12 items rated on a four‐point Likert‐type response scale from 0–3 (never, at normal level, higher than normal level, and much higher than normal level). Scores range between 0–36, and a score higher than 14 indicates undesirable general health. This tool has been translated into Persian, and its reliability has been confirmed and reported at 0.87 using Cronbach's alpha method (Montazeri et al., 2003). Its Cronbach's alpha value was 0.73 in the present study. Kim et al.'s Turnover Intention Questionnaire was used to assess turnover intention (Kim & Leung, 2007). This questionnaire includes 15 questions scored on a five‐point Likert scale (1 = strongly disagree to 5 = strongly agree), and the sum of the scores varies between 15–75 with higher scores indicating higher turnover intention. The Persian version of the questionnaire has been validated in Iran and its Cronbach's alpha was 0.82 (Barzanji, 2013). The Cronbach's alpha value of the Turnover Intention Questionnaire in this study was 0.76. To assess job strain, the 25‐item Job Content Questionnaire (JCQ) with four dimensions was used. The dimensions selected for this study included psychological job demands (5 items), which assess mental effort, quantity of work and time constraints for one's work; decision latitude (9 items), which includes two separate but complementary subscales of "skill discretion" comprising six items that assess learning opportunities, development of creativity and skills, and diversity in job tasks, and "decision‐making authority" comprising three items that assess independence in personal work, and the ability to make decisions or participate in work‐related decisions; social support scale (8 items), which includes two subscales of “co‐worker support” and “supervisor support”; and job insecurity comprising 3 items that assess job stability and frequency of dismissals. The JCQ items were scored on a four‐point scale from 1 (strongly disagree or never) to 4 (strongly agree or often). The score of this questionnaire was calculated according to the JCQ user guide (Karasek, 1985). The Persian version of the JCQ (P‐JCQ) has been confirmed in Iran (Choobineh et al., 2011). Its Cronbach's alpha value in this study was 0.77. Data were analysed with SPSS v.22 statistical software using correlation, *t* test, analysis of variance, multiple regression and descriptive tests.

## 3 RESULTS

A total of 479 nurses participated in the study. The mean age and work experience of participants was 33.43 (*SD*: 6.77) years and 9.19 years (*SD*: 6.42), respectively. The majority of the participants were female (61.6%), nurses (93.1%), married (78.1%), and had a bachelor's degree (84.8%). The majority of the nurses (45.5%) were working in the general medicine department and earning an annual salary of between US$ 2100–2550 (34.8%). The study results showed that gender was positively correlated with turnover intention (*p* = .001; Table 1).

**TABLE 1:** Demographic characteristics of nurses who worked during an COVID‐19 outbreak (*n* = 479)

Variables	Mean	*SD*	*N*	%	*p* value
Age	33.48	6.77			*r* = −0.26 *p* = .547
Gender					
Male			184	38.4	*t* = 3.472 *p* = .001
Female			295	61.6	
Work experience	9.19	6.42			*r* = 0.31 *p* = .500
Marital status					
Single			135	28.2	*t* = −1.571 *p* = .117
Married			344	71.8	
Educational status					
Associate			42	8.8	*F* = 1.429 *p* = .241
Bachelor			406	84.8	
Master or PhD			31	6.5	
Work position					
Nurse			446	93.1	*F* = 1.417 *p* = .244
Staff			17	3.5	
Supervisor			16	3.3	
Department					
ED^a^			107	22.3	*F* = 1.920 *p* = .125
General			218	45.5	
ICU			70	14.6	
EMS^b^			84	17.5	
Annual income (US$)					
1,200–1,650			56	11.7	*F* = 0.120 *p* = .948
1,650–2,100			154	32.2	
2,100–2,550			184	38.4	
2,550<			85	17.7	

^a^ Emergency Department.

^b^ Emergency Medicine Service.

The mean score of turnover intention was 41.73, ranging from 15–75; the PTSD score ranged from 0–88 with a mean of 36.96; the mean score of general health was 13.33, ranging from 0–36; and the mean job strain was 1.04, ranging from 0.5–2 (Table 2).

**TABLE 2:** Descriptive statistics of the study variables (*n* = 479)

Variable	Mean	*SD*	Min	Max	Range
Turnover intention	41.73	12.11	15	75	15–75
PTSD	36.96	16.34	00	88	00–88
General health	13.33	4.34	3	27	00–36
Job content					
Decision latitude	68.56	7.97	38	92	24–96
Job demand	35.60	5.46	18	48	12–48
Social support	21.68	4.12	8	32	8–32
Job insecurity	5.97	1.48	3	11	3–12
Job strain	1.04	0.18	0.56	2	0.5–2

Pearson's correlation coefficient showed that the mean scores of PTSD, general health, job demand, job strain, and job insecurity had a positive relationship with nurses’ turnover intention. A negative and significant relationship was found between the mean score of social support and nurses' turnover intention (*p* < .01; Table 3).

**TABLE 3:** Correlations among the study variables (*n* = 479)

Variable	1	2	3	4	5	6	7	8
Turnover intention	1.00							
PTSD	0.348**	1.00						
General health	0.311**	0.286**	1.00					
Decision latitude	0.016	0.050	0.893	1.00				
Job demand	0.298**	0.114*	0.029	0.215**	1.00			
Social support	−0.348**	−0.111*	−0.070	0.229**	0.257**	1.00		
Job insecurity	0.269**	0.136**	0.234**	−0.043	0.220**	0.232**	1.00	
Job strain	0.271**	0.077	0.040	−0.480**	0.741**	−0.357**	0.229**	1.00

Note

1 = Turnover intention, 2 = PTSD, 3 = General health, 4 = Decision latitude, 5 = Job demand, 6 = Social support, 7= Job insecurity, 8 = Job strain.

* *p* < .05,

** *p* < .01.

Multiple linear regression predicted job stressors in nurses that affect turnover intention. Job strain (*β* = 0.624, *p* = .013) had the greatest impact, followed by decision latitude (*β* = 0.445, *p* = .011), PTSD (*β* = 0.238, *p* < .001), and general health (*β* = 0.209, *p *< 0.001). In addition, married status (*β* = 0.111, *p* = .009) was positively correlated with turnover intention, while social support (*β* = −0.243, *p* < .001), gender (*β* = −0.131, *p* = .002) and work position (*β* = −0.084, *p* = .038) were negatively correlated with turnover intention. These variables explained 35% of the variance in turnover intention among nurses in this study (Table 4).

**TABLE 4:** Multiple linear regression predicting turnover intention (*n* = 479)

Variables	*B*	*SE*	Beta	*T*	Sig	*R*	*R* square
(Constant)	−19.826	18.670		−1.062	0.289	0.591^a^	0.350
Age	−0.010	0.160	−0.006	−0.065	0.948		
Gender	−3.263	1.028	−0.131	−3.173	0.002		
Marital status	2.976	1.132	0.111	2.629	0.009		
Educational status	0.162	1.218	0.005	0.133	0.894		
Work position	−1.797	0.862	−0.084	−2.085	0.038		
Work experience	−0.071	0.164	−0.038	−0.430	0.667		
Department	0.806	0.488	0.066	1.653	0.099		
PTSD	0.177	0.030	0.238	5.904	0.000		
General health	0.582	0.113	0.209	5.162	0.000		
Decision latitude	0.677	0.264	0.445	2.562	0.011		
Job demand	−0.813	0.501	−0.367	−1.622	0.106		
Social support	−0.714	0.124	−0.243	−5.771	0.000		
Job insecurity	0.484	0.353	0.059	1.371	0.171		
Job strain	41.425	16.660	0.624	2.486	0.013		

^a^ Dependent variable: Turnover intention.

## 4 DISCUSSION

Nurses are considered to be the most valuable asset in healthcare systems (Madlabana et al., 2020). Developing and retaining skilled and motivated health staffs is critical to improving the quality of healthcare. The aim of the present study was to identify the predictors of nurses’ turnover intention based on psychosocial factors in nurses of Ardabil pre‐hospital emergency and educational and medical centres during the COVID‐19 outbreak. In the present study, the mean turnover intention among 479 nurses was 41.73 (scores ranged from 15–75). The results of a study conducted by Hariri showed that the mean turnover intention among nurses in Tehran was 3.35 (scores ranged from 1–5) in conditions where infectious diseases were not common (Hariri et al., 2012). Chen et al. conducted a study in China in 2018 and revealed that the mean turnover intention among nurses was 15.50 (scores ranged from 1–24; Chen et al., 2018). A study conducted by Cai et al. in China showed a mean turnover intention among nurses of 3.39 (scores ranged from 1–5; Cai & Zhou, 2009). Jung et al. reported that in South Korea (Jung et al., 2020), the mean score of turnover intention under the conditions of the SARS outbreak was 16.3 (scores ranged from 4–20). Santos et al. conducted a study in the Philippines (Labrague & De los Santos, 2021) and reported that during the COVID‐19 outbreak, turnover intention was 2.87 (scores ranged from 1–5). Evidence suggests that the mean turnover intention among nurses in different communities varies, depending on the prevalence of viral diseases. These differences might be attributable to multiple definitions of the phenomenon of turnover intention or to the differences in research settings in the mentioned studies. The results of the present study revealed that the mean of turnover intention was significantly correlated with the variable of gender with a higher turnover intention seen in men compared to women. Conversely, the study conducted by Lee (2019) in South Korea and that conducted by Sokhanvar et al. (2018) did not show a significant relationship between turnover intention and gender, which might be attributable to the cultural context of Iran, where men have more difficulty tolerating a work setting that is stressful and out of control, for example during an outbreak of an infectious disease, and they are more likely to suffer from stress.

The research results revealed that turnover intention had a positive and significant correlation with PTSD and general health. These results are in line with the results of a study by Jung et al. conducted during the outbreak of MERS (Jung et al., 2020). Because nurses are directly involved in caring for infected patients, they are at a higher risk of developing COVID‐19 than other groups in a community. This issue can cause a sense of panic, fear of being infected, or a fear of transmitting the infection to others such as family members and friends. Moreover, worries about the outbreak of the disease, including the increasing number of patients, providing health services to patients infected with COVID‐19, maintaining social distancing and quarantining communities, can exacerbate fear among nurses and affect their mental, emotional and functional health (Labrague & De los Santos, 2021). In fact, undesirable general health affects turnover intention by increasing job stress. However, having good general health is one of the inhibitors of the adverse effects of job stress (PEIMAN et al., 2013).

The results of the present study also showed that turnover intention had a positive and significant correlation with job demand and job insecurity. These results are in line with those of the study conducted by Barzideh et al. Job stress is a result of high job demand and low decision latitude on job conditions and job insecurity. Job demand includes both physical and psychological demands (Barzideh et al., 2013). Job characteristics as a source of stress can play a major role in people's stress levels. High independence and low complexity at work are associated with lower anxiety and complaints, and conversely, low independence and high complexity at work are associated with higher anxiety and complaints job characteristics as a source of pleasure (high control and low complexity) can reduce job stress and create a sense of competence in a person. However, low control and high complexity increase job stress and reduce self‐esteem. The results of the Väänänen study showed that a high‐quality relationship with supervisor support may reduce the impact of job demand (e.g. overtime, emotional and physical demands) on job stress, because appreciation and support from supervisors put demands into perspective. Supervisor support may also help an employee cope with job demands, facilitate performance and act as a protector against adverse conditions. Administrative support reflects the appreciation and support of the organization, which may help employees cope with job demand and illness (Väänänen et al., 2003). Thus, the positive relationship between job demand and turnover intention and the negative relationship between job control and turnover intention seem to be logical. The mean score of turnover intention in nurses revealed a significant negative correlation with social support. In other words, increasing co‐worker and supervisor support decreases turnover intention in nurses. This result is in line with the results of similar studies conducted in this area (Alipour et al., 2018; Barzideh et al., 2013; Jung et al., 2020). This support can be provided by a supervisor or by co‐workers. When people feel that they are supported at their workplace, their turnover intention decreases. In addition to personal and personality traits, the social support network plays a key role in reducing response to job stress. Maintaining health and preventing stress are other main functions of social support (Brouwers et al., 2001), an issue the results of the present study confirmed. Social support at the workplace, including supervisor and co‐worker support, has also been found to play a moderating role on the effects of high stress on well‐being and related health outcomes (Cohen & Wills, 1985).

The present results revealed that job stress, decision latitude, PTSD and general health had the highest power in predicting turnover intention during the outbreak of COVID‐19. Nurses with high levels of work stress are at a higher risk of turnover intention (DeTienne et al., 2012). The results of a study conducted by Nissly et al. indicated that people with higher job stress were more likely to quit their jobs, while people with more social support were less likely to think about quitting their jobs. The results also showed that turnover intention had a significant and positive relationship with the level of organizational stress and conflict between work and family among participants. Compared to the power of organizational stress, the impact of work–family conflict was small. It was also found that social support predicts turnover intention (Nissly et al., 2005). Outcomes of human resource management for staffs with organizational support (supervisor support), desirable job conditions and development of their job goals are effective ways to reduce stressors and ultimately reduce turnover intention. The results of Tetteh et al. showed that job stress is a significant mediating variable that affects employee turnover intention. Employees who experience high levels of work‐related stress are more likely to seek alternative jobs. Conversely, a high level of perceived organizational support leads to a reduction in stress and related illnesses and an increase in employee retention (Tetteh et al., 2020). Organizations that provide more support to employee well‐being are more likely to reduce stress and ultimately increase the employees’ intention to stay in the organization. Organizations that can understand the reasons and factors influencing employees’ turnover intentions will be able to apply effective policies and practices to retain human resources before employees leave the organization.

### 4.1 Research limitations

Because the results of different hospital departments were not investigated in this study due to their large number and variety, the present results cannot be generalized to all hospital departments. Studies with larger sample sizes in any hospital departments can eliminate this limitation. Moreover, because this study was conducted only on nurses in Ardabil, one should proceed with caution in generalizing the results to nurses in other regions. Given the special requirements of the nursing job and fatigue, work pressure and time constraints in completing the questionnaires during the outbreak of the COVID‐19 disease, it is recommended that a similar study be conducted after the disease has been completely controlled to access a sample population outside the hospital setting, to provide further explanations on cooperation and completion of questionnaires, and to compare the results under different conditions.

## 5 CONCLUSION

The results of the present study revealed that job stressors during the COVID‐19 outbreak shape nurses' attitudes towards their jobs and can cause turnover intention in nurses. Thus, these factors should be identified and managed so as to prevent turnover intention in such critical situations. Employee support systems should be discussed at the administrative, organizational and national levels. Most importantly, coping strategies to reduce stress during outbreaks of infectious disease through the support of co‐workers, caregivers and supervisors should be actively used by nurses to reduce their turnover rates. Infectious disease and occupational health professionals should take steps to develop and implement coping management strategies with the aim of reducing job strain and turnover intention caused by outbreaks of infectious diseases and prepare programmes for caregivers to provide adequate support to nurses.

## CONFLICT OF INTEREST

Each of the authors has read the submission and declare that they have no conflicts of interest related to the study.

## AUTHOR CONTRIBUTIONS

All authors contributed to the design of the study and data collection. Alireza Mirzaei drafted the manuscript after data analysis and Aghil Habibi Soola revised it critically for important intellectual content. All the other co‐authors also made significant contributions to the revision of the manuscript.

## ETHICAL APPROVAL

The protocol of this study was approved by Research Ethics Committee, Ardabil University of Medial Sciences (IR.ARUMS.REC. 1399.035).

## Data Availability

The data that support the findings of this study are available from the corresponding author upon reasonable request.

## ENDNOTE

^1^
Iran's Ministry of Health divided the country into so‐called white, yellow, and red zones depending on fatality rate and extent of outbreak of COVID‐19. Zones with more than three COVID‐19 cases per 100,000 population are categorized as red, while white zones are areas with the lowest threat with less than one (Worldaware, June 11, 2020).
